# Depression, anxiety, and stress among COVID-19 patients in South Sinai, Egypt: prevalence and associated predictors

**DOI:** 10.1038/s41598-023-45775-z

**Published:** 2023-11-06

**Authors:** Basma Khairy Gad, Mostafa Ahmed Arafa, Ashraf Farouk Attia, Ahmed Hassanin Farahat, Marwa Shawky Abdou

**Affiliations:** 1 Ministry of Health and Population, Preventive Medicine Sector, South Sinai, Egypt; 2 Department of Epidemiology, High Institute of Public Health, Alexandria University, 165 El-Horeya Road, Al Ibrahimeyah Qebli WA Al Hadrah Bahri, Bab Sharqi, Alexandria, Egypt; 3 Department of Epidemiology, Military Medical Academy, Heliopolis, Cairo, Egypt

**Keywords:** Diseases, Infectious diseases

## Abstract

Infectious diseases trigger fear and anxiety among patients leading to disturbance in psychological health of patients. Psychological symptoms were found during SARS-COV-1 epidemic which raise the curiosity about their presence with SARS-COV-2 infection. The current study aimed to estimate the prevalence and severity of psychiatric disorders (depression, anxiety, and stress) among COVID-19 patients and their associated significant predictors. A cross-sectional study was conducted among 382 patients infected with COVID-19 in South Sinai governorate, Egypt, during the period June 2021 through January 2022. Patients with positive PCR test for COVID-19 were included if no more than 6 months have passed after being isolated in the hospital or at home. The patients were being contacted after being cured from COVID-19. The Arabic version of the Depression Anxiety Stress was used to assess the psychological status of patients. Multivariate linear regression analysis was done to detect the predictors of psychiatric symptoms among patients. A total of 382 participants with mean age of 41.5 ± 15.0 years old, of whom 72.5% were males were included in the study. 91.6% of participants had all the three studied psychological disorders; depression, anxiety and stress with most of participants had either severe or extremely severe conditions (13.9 and 75.7 for anxiety, 22.8 and 46.3% for depression, 38.5 and % 19.6 for stress, respectively). Healthcare workers had higher prevalence rates of depression, anxiety and stress. In bivariate analysis, educational level, HCWs and visiting healthcare facility were significantly affecting DASS scores. In linear regression analysis, hospital admission was the main predictor of the three psychological disorders. In Conclusion, majority of patients affected with COVID-19 suffered from symptoms of anxiety, depression and stress within six months after being infected. Hospital admission was found to be the main predictor of the presence of psychiatric disorders with prolonged recovery time from COVID-19 infection. More attention should be paid to COVID-19 patient’s mental health as psychological care and presence of psychiatric in the isolation hospitals should be considered.

## Introduction

During the recent pandemic, Egypt was one of five countries reporting the highest number of COVID-19 cases in Africa, with a total of 516,023 confirmed cases and 24,830 deaths from January 3, 2020 till December 6, 2023^1^. By December 2020, the percentage of COVID-19 cases in the South Sinai governorate was estimated to be 0.5% of all cases in Egypt^2^.

Infectious diseases often trigger waves of heightened fear and anxiety that can have serious negative effects on the behavior and psychological well-being of many people in a community^3^. Studies done at the end of the SARS-COV-1 pandemic revealed many COVID-19 patients to have had depressive symptoms while they were infected with the virus^4^. Severe infections and inflammatory processes can cause delirium with a variety of psychiatric symptoms and encephalopathy; such symptoms have been reported to be found among SARS-COV-2 positive patients, but the evidence is still scarce, and the neurotropic potential of SARS-COV-2 requires elucidation^5^. The risk of psychiatric disorders among these patients could be due to the virus directly or indirectly inducing a massive cytokine response in the brain^6^.

Psychiatric symptoms that occur during COVID-19 infection or following recovery may persist in the ensuing weeks, months, or longer. One of these symptoms is generalized anxiety disorder (GAD) which is characterized by excessive and persistent worrying that is hard to control and occurs more days than not for at least six months. Stein performed a systematic review examining psychiatric disorders in patients hospitalized for severe acute respiratory syndrome (SARS) or Middle East respiratory syndrome (MERS) and revealed a prevalence of anxiety, depression, and post-traumatic stress disorder ranging from 15 to 32% three to 46 months after recovery^7^.

Previous studies in this area have focused mainly on COVID-19-related mental health issues among the general population, medical staff, children, pregnant women and their husbands, people with mental illness, and individuals in self-isolation^8^, thus, very little is known about the psychological effects of the disease on patients with COVID-19, particularly in the South Sinai governate of Egypt. Because South Sinai is a tourism hub in Egypt, epidemics and infections there can have a significant effect on the movement of tourism and subsequently on local economy of the country. Thus, the aim of the current study was to estimate the prevalence and severity of psychiatric disorders (depression, anxiety, and stress) and their significant predictors among COVID-19 patients in South Sinai, Egypt.

## Methods

It was a cross-sectional study conducted in South Sinai Health Directorate and homes of patients with COVID-19 in South Sinai governorate, Egypt, during the period June 2021 through January 2022. From the records, patients with positive PCR test for COVID-19 were included if no more than six months have passed after being isolated in the hospital or at home. Patients were excluded if they are living outside or moved out of the Governorate. A sample size was calculated, based on a prevalence of depression and anxiety among COVID-19 patients of 32%^9^ and 5% confidence limit, the minimum required sample size at 95% confidence level was calculated to be 334 patients. All of them were recruited from the list of confirmed patients in the records. They were selected through systematic random sampling technique. A sampling frame was created from the records, the first patient was selected randomly, then every third patient was selected to be included in the study, those who did not meet the inclusion criteria were skipped and the following patient was selected. Those who refused, out of reach, living outside the governorate, and those who are not Egyptians were skipped from the list and being replaced with another patient. All selected patients were contacted by phone and paid a visit at home after being discharged from the hospital. The objectives and the rationale of the study were fully explained to all of them.

There are 8 hospitals in the Governorate. All of them are sending the information about COVID-19 patients immediately and regularly to the Directorate of the Ministry of Health and population^2^ at South Sinai Governorate, which is responsible for collecting and manipulation the data about the patients. The data was selected from the main and central records at the MOHP.

A predesigned structured interviewing questionnaire was used to collect the following data from patients: socio-demographic data, medical history, and COVID-19 infection data. In addition to smoking, alcohol or substance use, and history of psychiatric disorders.

### Psychological test

The Arabic Version of the Depression Anxiety Stress Scale (DASS-42), was used to assess the psychological status of patients^10^. The score was originally developed by the University New South Wales, Australia^11^.

The DASS-42 is a 42 item self-report scale designed to measure the negative emotional states of depression, anxiety and stress. The principal value of the DASS in a clinical setting is to clarify the symptoms of emotional disturbance, as part of the broader task of clinical assessment. It has been shown to be a valid and reliable measure of the dimensions of depression, anxiety, and stress separately but also taps into a more general dimension of psychological distress. The response of each question varies from 0 (did not applied to me at all) to 3 (applies to me very much to me or most of the time). Sum scores for the total DASS-total scale range between 0 and 126, where normal individuals attained scores 0–77 while those with severe psychological distress attained score ranged from 98 to 126. Each subscale has its own cut-offs and could be classified as; normal, mild, moderate, seveer and extremely sever psychological distress, i.e. normal levels for depression, anxiety and stress ranged from 0 to 9 & 0–7 and 0–14 points respectively. While severe levels ranged from 21 to 27 & 15–19 and 26–33 points respectively. Above these corresponding scores are considered extremely severe levels.

### Statistical analysis

The collected data was coded and analyzed using the SPSS software (Armonk, NY: IBM Corp version 25.0)^12^. The quantitative variables were expressed using mean ± SD while categorical variables were described by counts (%). Kolmogorov–Smirnov test was done to detect the normal distribution of the data. Kruskal–Wallis’ test and independent t test were used to estimate the relation between continuous variables. Linear regression models using all the variables were conducted to estimate the significant predictors affecting the presence of psychiatric disorders. Statistical significance was considered when *p* < 0.05.

### Ethics approval and consent to participate

This study was approved by the Ethics Committee of the High Institute of Public Health, Alexandria University, Egypt. The study was performed in accordance with the international ethical guidelines of the Declaration of Helsinki^13^. All participants were informed with the purpose and nature of the study, the privacy and confidentiality of data, and participation was voluntary. An informed consent was obtained from all participants prior the start of the study after explanation the study protocol and assurance of anonymity that all subjects were going to be represented by codes rather than their names.

## Results

The study included 382 participants, their mean age was 41.5 ± 15 years old, 277 (72.5%) were males and 27.5 were females. Majority of patients (98.2%) were Egyptians and the remaining 1.8% were foreigners. 73.8% were married, while singles constituted 22.3%, divorced 0.8% and widowed 3.1%. Eighty eight percent had a private residence. Sixty percent of participants had a university degree, while illiterates constituted only 6.0%. Less than one forth (23.8%) were health care workers, (physicians, nurses, lab technicians, radiology technicians). 65.4% of patients were admitted to hospital with a mean duration of hospitalization of 7.95 ± 6.976 days, mean time of recovery of 17.56 ± 5.89 days and mean duration if stay in isolation place of 13.59 ± 5.05 days. 26.4% of patients were treated with hydroxychloroquine with a mean duration of treatment of 6.9 ± 4.91 days. Only 26.4% of patients had a history of visiting a medica facility and 22.3% had time for physical activity.

Table 1 illustrated the distribution of DASS score among the study participants. Anxiety was exceedingly frequent among study participants; extremely severe (75.7%) and severe (13.9%), followed by depression; extremely severe (46.3%) and severe (22.8%), the least frequent prevalent symptom among the participants was stress, extremely severe (19.6%), severe (38.5%). The mean scores of DASS were 24.9 ± 8.6, 24.04 ± 7.7 and 26.1 ± 8.1 for depression, anxiety and stress, respectively.

**Table 1:** Distribution of study sample as regards DASS SCORE.

DASS SCORE	Study sample (n = 382)	
	No	%
Depression grades		
Normal	18	4.7
Mild	24	6.3
Moderate	76	19.9
Severe	87	22.8
Extremely severe	177	46.3
Depression score	24.9 ± 8.6	
Anxiety grades		
Normal	12	3.1
Mild	6	1.6
Moderate	22	5.8
Severe	53	13.9
Extremely severe	289	75.7
Anxiety score	24.04 ± 7.7	
Stress grades		
Normal	33	8.6
Mild	30	7.9
Moderate	97	25.4
Severe	147	38.5
Extremely severe	75	19.6
Stress score	26.1 ± 8.1	
Total DASS score	75.04 ± 22.9	

Combined mental disorders was calculated by adding all three categorical variables and it was found that, the majority of the studied patients (91.6%) had all the three psychological disorders; depression, anxiety and stress, while only 2.9% of them had no disorders, Table 2.

**Table 2:** Distribution of study sample as having psychological disorders.

	Study sample (n = 382)	
	No	%
No disorder	11	2.9
One disorder	8	2.1
Two disorders	14	3.7
All three disorders	382	91.6

**Figure 1:**
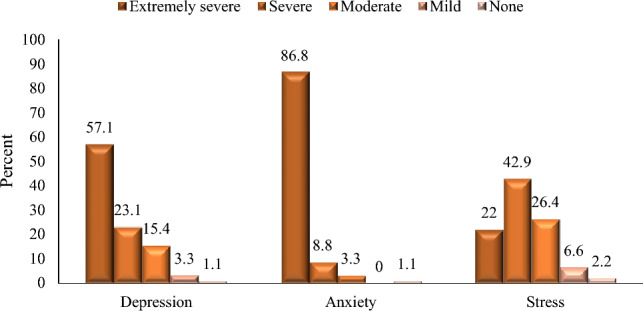
HCWs affection with depression, anxiety and stress.

We looked at the prevalence of DASS among HCWs alone. 57.1% of HCWs had extremely severe depression, 23.1% had severe depression, 15.4% had moderate depression and 3.3% had mild depression. Regarding anxiety grades, the majority of HCWs (86.8%) had extremely severe anxiety, followed by 8.8% had severe anxiety and 3.3% had moderate anxiety. Stress grades showed a variable percentage among HCWs: 22% extremely severe stress, 42.9% severe, 26.4% moderate, 6.6% mild, and 2.2% had no stress, Fig. 1.

Table 3 shows that the educational level (p = 0.034), being a healthcare worker (p = 0.003), regular visiting the medical care facility (p < 0.001) and not practicing physical activity (p = 0.03) were significantly associated with depression score. Being a health care worker and visiting a medical care facility are significantly associated with anxiety, (p = 0.03 and p = 0.01 respectively). The significant parameters associated with the stress score were educational level (p < 0.001), being a healthcare worker (p = 0.004), regular visiting the medical care facility (p = 0.007), treatment with hydroxychloroquine (p = 0.02) and not practicing physical activity (p = 0.029).

**Table 3:** Significant variables associated with DASS items.

	Study sample (n = 382)		Depression	P	Anxiety	P	Stress	P	Total	P
	No	%								
Educational level										
University or higher	232	60.7	25.72 ± 8.3	0.034* ^A^	24.64 ± 7.6	0.15	27.46 ± 7.7	< 0.001*^B^	77.82 ± 22.0	0.006* ^C^
Secondary	114	29.8	24.13 ± 9.0		23.22 ± 7.5		24.61 ± 8.6		71.96 ± 23.9	
Preparatory	7	1.8	24.6 ± 11.3		25.0 ± 10.2		24.7 ± 10.1		74.29 ± 31.0	
Primary	6	1.6	25.33 ± 5.0		25.0 ± 10.9		23.83 ± 4.0		74.17 ± 13.1	
Illiterate	23	6.0	20.22 ± 8.2		21.52 ± 8.1		20.96 ± 7.2		62.69 ± 22.0	
Occupation										
Health care workers	91	23.8	27.23 ± 7.2	0.003*	25.53 ± 6.4	0.035*	28.23 ± 6.4	0.004*	80.98 ± 18.4	0.004*
Non-health care workers	291	76.2	24.15 ± 8.8		23.58 ± 8		25.45 ± 8.5		73.18 ± 23.87	
History of visiting a medical care										
Yes	101	26.4	27.43 ± 6.9	< 0.001*	25.70 ± 6.0	0.01*	27.97 ± 6	0.007*	81.1 ± 17.7	0.002*
No	281	73.6	23.97 ± 8.9		23.44 ± 8.0		25.44 ± 8.6		72.86 ± 24.2	
Treatment with hydroxychloroquine										
Yes	185	48.4	25.6 ± 7.6	0.151	24.3 ± 6.3	0.5	27.1 ± 7.0	0.02*	77.0 ± 18.6	0.1
No	197	51.6	24.2 ± 9.4		23.8 ± 8.8		25.2 ± 9		73.2 ± 26.2	
Time for physical activity										
Yes	85	22.3	23.11 ± 9.2	0.03*	23.29 ± 8.6	0.3	24.4 ± 9	0.029*	70.8 ± 25.9	0.054
No	297	77.7	25.39 ± 8.3		24.25 ± 7.4		26.5 ± 7.7		76.24 ± 21.8	

Data are presented as mean ± SD, analysis were done using Kruskal Wallis and independent t test.

Pairwise comparison was done as A; sig detected between (illiterate vs university, p = 0.021*); B; sig detected between{(illiterate vs university, p = 0.0001*) & (secondary vs university, p = 0.02*)}; C; sig detected between (illiterate vs university, p = 0.007*).

In the linear regression model only two variables were the significant predictors of depression; being a HCW (p = 0.048), hospital admission (p = 0.005), time to recovery (p = 0.008) and duration of hospital stay (p = 0.007), the model explains 18.7% of variability in depression score, while for anxiety and stress; hospital admission was the only significant predictors for anxiety and stress; (p = 0.002, p < 0.001, respectively). Both models explain 10.7% and 14% of variability in anxiety and stress respectively, Table 4.

**Table 4:** Multivariate linear analysis to assess the independent contribution of different sociodemographic and clinical characteristics of participants to DASS score.

Predictors	Unstandardized coefficients, B std. error		Standardized coefficients, Beta	t	Individual predictors sig	95% confidence interval
Depression						
Constant	15.709	3.305		4.753	< 0.001*	9.193–22.226
Age	−0.062	0.038	−0.119	−1.613	0.108	−0.137 to 0.014
Male^a^	−0.032	1.249	−0.002	−0.026	0.980	−2.494 to 2.430
Occupation^b^	2.842	1.428	0.131	1.990	0.048*	0.026 to 5.658
Hospital admission^c^	6.547	2.307	0.212	2.838	0.005*	1.999 to 11.095
Time of recovery	0.373	0.139	0.256	2.695	0.008*	0.100 to 0.646
Duration of hospital stay	−0.310	0.114	−0.246	−2.727	0.007*	−0.533 to −0.086
Duration of stay in the isolation place	0.063	0.124	0.038	0.505	0.614	−0.182 to 0.307
Time for physical activity^d^	−0.944	1.467	−0.044	−0.644	0.520	−3.836 to 1.948
Anxiety						
Constant	17.793	3.419		5.204	< 0.001*	11.046 to 24.541
Age	−0.037	0.040	−0.076	−0.914	0.362	−0.116 to 0.043
Male^a^	−1.472	1.331	−0.080	−1.106	0.270	−4.099 to 1.155
Occupation^b^	1.594	1.669	0.071	0.955	0.341	−1.7 to 4.889
Hospital admission^c^	6.787	2.171	0.256	3.126	0.002*	2.502 to 11.07
Time of recovery	0.131	0.139	0.092	0.937	0.350	−0.145 to 0.406
Duration of hospital stay	−0.064	0.110	−0.053	−0.585	0.559	−0.282 to 0.153
Duration of stay in the isolation place	0.010	0.128	0.007	0.077	0.939	−0.242 to 0.262
Time for physical activity^d^	−0.886	1.713	−0.040	−0.517	0.605	−4.267 to 2.49
Stress						
Constant	19.935	3.490		5.713	< 0.001*	13.048 to 26.822
Age	−0.059	0.041	−0.117	−1.439	0.152	−0.14 to 0.022
Male^a^	−0.632	1.358	−0.033	−0.465	0.642	−3.3 to 2.04
Occupation^b^	1.809	1.704	0.077	1.061	0.290	−1.55 to 5.17
Hospital admission^c^	8.031	2.216	0.291	3.624	< 0.001*	3.658 to 12.4
Time of recovery	0.137	0.142	0.093	0.966	0.336	−0.143 to 0.42
Duration of hospital stay	−0.101	0.112	−0.080	−0.901	0.369	−0.323 to 0.12
Duration of stay in the isolation place	−0.027	0.130	−0.017	−0.210	0.834	−0.285 to 0.23
Time for physical activity^d^	−1.245	1.748	−0.054	−0.712	0.477	−4.695 to 2.2

*; Significant (p ≤ 0.05).

^a^Ref; female.

^b^Ref; non HCWs.

^c^Ref; not admitted.

^d^Ref; no time for physical activity.

## Discussion

Reducing psychological stress is an essential aspect of COVID-19 treatment strategies. Since its appearance, COVID-19 has had a significant impact on people's health and quality of life worldwide^14^. Both the infected and non-infected could be at risk of mental health problems as a result of issues such as widespread anxiety, social isolation, healthcare, and other essential workers’ stress, and unemployment and financial difficulties^15–17^. Other experiences might be specific to individuals infected with the virus, such as concern over the outcome of their illness^18^.

While many studies have examined the physical and financial effects of the coronavirus, few have examined the mental health care needs of COVID-19 patients^18^. Thus, this study was conducted to determine the prevalence of emotional disturbance mental health symptoms as well as the probable correlates of these symptoms among COVID-19 patients in the South Sinai governorate of Egypt. The results revealed a high prevalence of anxiety, depression, and stress among COVID-19-infected patients, with 75.7% having extremely severe anxiety, 46.3% having extremely severe depression, and 19.6% having extremely severe stress.

A cross-sectional study conducted by Li et al.^19^ in China investigated the prevalence of anxiety and depression among COVID-19 hospitalized patients during the pandemic and found a mean anxiety score of 6.69 ± 5.01 and a mean depression score of 8.27 ± 5.35. Healthcare workers (HCWs) were found to be most likely to experience depression, anxiety, or stress as COVID-19 patients, with 80.2% having extremely severe or severe depression, 86.8% having extremely severe or severe anxiety, and 64.9% having extremely severe or severe stress.

A cross-sectional study in a university hospital in Egypt examined a sample of 270 HCWs employed in COVID-19 isolation units; the DASS revealed a significant frequency of depression disorders, with 28.1% of the HCWs having mild to moderate depressive symptoms and 64.8% having severe symptoms^20^. Another study conducted in Egypt found a high prevalence of severe depressive symptoms among Egyptian physicians, with 63% having severe or extremely severe depressive symptoms, 77.6% having extremely severe anxiety, and 72% experiencing stress^21^. In Jordan, a study conducted by Naser et al.^22^ found a high prevalence of depressive (78.1%) and anxiety (70.8%) symptoms among healthcare professionals. In 2003, a study using the DASS-21 during the SARS outbreak found that 93% of patients had experienced depressive symptoms, with 65% having severe or extremely severe symptoms. Nearly 90% of HCWs who had dealt with SARS patients during the outbreak experienced psychological symptoms. Thus, viruses similar to COVID-19 can be linked to the presence of mental health symptoms among patients and their caregivers^23^.

HCWs are the first line of defense against highly infectious diseases with unclear outcomes. With a lack of infection control measures and protective equipment, HCWs face a significant amount of stress that may cause them to have a higher prevalence of psychological disorders than ordinary patients^24^. Li et al. conducted a study in China that demonstrated a substantial positive correlation between depression and anxiety scores using the Hospital Anxiety and Depression Scale (HADS); these findings were similar to ours in which the presence of depressive symptoms increased the risk of anxiety and stress symptoms among COVID-19 patients^19^.

Our linear regression results revealed that hospital admission was the main predictor of depression, anxiety and stress and the duration of hospital stay with extended recovery time from COVID-19 symptoms were significantly affecting appearance of depression after infection. Previous studies have also found psychological problems to be linked to prolonged hospitalization for multiple diseases^18,25^. Others have linked longer hospital stays with higher levels of anxiety and depression in COVID-19 patients^26,27^. Elgohary et al.^20^ identified other predictors affecting the presence and severity of psychological disorders among COVID-19 patients, including young age, decreased sleep hours, being female, a past history of a psychiatric disease, fear of COVID-19 infection in themselves or their relatives, and fear of death from COVID-19 for themselves or their relatives. Similarly, Khanal et al.^24^ reported that females, divorced people and university students with history of chronic disease and high-income earners (≥ 1500 JD) were at greater risk of developing anxiety during their infection with COVID-19.

Patients with longer disease duration were shown to have a more depressive attitude about their condition. Effect of steroid use and grade of dyspnea on development of moderate or severe post-COVID depression and showed that higher grades of dyspnea were associated with higher probability of development of moderate or severe post-COVID depression^28^.

The present study faced some limitations including being single-centered which affect the representation and generalization of results among all patients infected with COVID-19. Other limitations included the fear of patients to admit having problems during COVID-19 infection and Arabic version of DASS has a lot of similar questions which confuse the patients upon answering.

## Conclusion

The present work found that the majority of COVID-19 infected patients suffered from anxiety, depression and stress following infection. The three studied disorders were found to interact together among most of the participants. Hospital admission is the main predictor led to occurrence of psychiatric disorders with prolonged time taken by the patients to recover from COVID-19 infection. Mental illness is one of the most important illnesses should be noticed in patients. Being infected, isolated or having symptoms for a long period of time will affect the mental status of patients. COVID-19 itself might have an impact on the mental status of patients. More attention should be paid to these patient’s mental health so psychological care and presence of psychiatric in the isolation hospitals can be considered.

## Data Availability

All data generated or analyzed during this study are included in this article and can be requested from the corresponding author.
